# High Tidal Volume Induces Mitochondria Damage and Releases Mitochondrial DNA to Aggravate the Ventilator-Induced Lung Injury

**DOI:** 10.3389/fimmu.2018.01477

**Published:** 2018-07-03

**Authors:** Jin-Yuan Lin, Ren Jing, Fei Lin, Wan-yun Ge, Hui-jun Dai, Linghui Pan

**Affiliations:** Department of Anesthesiology, The Affiliated Tumor Hospital of Guangxi Medical University, Nanning, China

**Keywords:** ventilator-induced lung injury, toll-like receptor 9/myeloid differentiation factor 88/nuclear factor-κB signaling pathway, mitochondrial DNA, mitophagy, mitochondrial damage

## Abstract

**Objective:**

This study aimed to determine whether high tidal volume (HTV) induce mitochondria damage and mitophagy, contributing to the release of mitochondrial DNA (mtDNA). Another aim of the present study was to investigate the role and mechanism of mtDNA in ventilator-induced lung injury (VILI) in rats.

**Methods:**

Rats were tracheotomized and allowed to breathe spontaneously or mechanically ventilated for 4 h. After that, lung injury was assessed. Inhibition of toll-like receptor 9 (TLR9), named ODN2088, was used to determine the involvement of TLR9/myeloid differentiation factor 88 (MyD88)/nuclear factor-κB (NF-κB) signaling pathway in VILI. The mitochondrial damage and release of mtDNA were assessed. Pharmacological inhibition of mtDNA (chloroquine) was used to determine whether mtDNA trigger inflammation *via* TLR9 in VILI. EDU-labeled mtDNA deriving from mitophagy was assessed by immunofluorescence. The role of mitophagy in VILI was shown by administration of antimycin A and cyclosporine A.

**Main results:**

Rats subjected to HTV showed more severe pulmonary edema and inflammation than the other rats. The decreased expression of TLR9, MyD88, and NF-κB were observed following the use of ODN2088. Mechanical ventilation (MV) with HTV damaged mitochondria which resulted in dysfunctional ATP synthesis, accumulation of reactive oxygen species, and loss of mitochondrial membrane potential. Moreover, the results of distribution of fluorescence in rats upon HTV stimulation indicated that mtDNA cleavage was associated with mitophagy. The expression levels of mitophagy related genes (LC3B-II/LC3B-I, PINK1, Parkin, and mitofusin 1) in animals ventilated with HTV were significantly upregulated. Administration of antimycin A aggregated the histological changes and inflammation after MV, but these effects were attenuated when administered in the presence of cyclosporine A.

**Conclusion:**

MV with HTV induces mitochondrial damage and mitophagy, contributing to the release of mtDNA, which may be induced VILI in rat *via* TLR9/MyD88/NF-κB signaling pathway.

## Introduction

Acute lung injury/acute respiratory distress syndrome is characterized as acute onset, intractable hypoxemia, and bilateral lung infiltration with high morbidity and mortality for hospitalized patients (1). Mechanical ventilation (MV) is a life-saving therapy for these patients. Paradoxically, inappropriate use of MV especially high tidal volume (HTV) can directly exacerbate lung injury, a syndrome termed as ventilator-induced lung injury (VILI). Increased pulmonary and vascular permeability, infiltrated inflammatory cells (2), activated immune responses (3), or oxidative stress (4) are the main contributing factors for VILI. The main mechanism of VILI includes volutrauma, barotrauma, atelectrauma, and biotrauma which consists of proinflammatory cytokines released, leukocytes recruited, and local inhibition of inflammatory processes (5).

Toll like receptors (TLRs) are well-known toll-like pattern recognition receptors that play an important role in the induction of innate immune and inflammatory responses. It is reported that TLR9 receptors are normally involved in initiating innate immune responding to damage-associated molecular patterns (DAMPs) (6). In our previous study, signaling involving TLR9 and myeloid differentiation factor 88 (MyD88) contributes to inflammation associated with the use of HTV ventilation for 4 h (7).

Mitochondria, under a variety of critical conditions, especially after damaged and dysfunctional, were engulfed and eliminated by selective autophagy (mitophagy) (8). Mitochondrial autophagy (mitophagy) is activated by mitochondrial permeability transitions, accumulation of reactive oxygen species (ROS), and loss of mitochondrial membrane potential (Δψm) with regulation of phosphatase and tensin homolog (PTEN) inducing putative kinase 1 (PINK1)/Parkin and mitofusin 1 (Mfn1) (8, 9). Mitochondria possess DNA similar to bacterial DNA, containing inflammatogenic unmethylated CpG motifs (10).

Mitochondrial DNA (mtDNA) is considered to be one of the mitochondrial DAMPs (11). Recently, a growing body of researches show that mtDNA triggers the immune response directly *via* the activation of TLR9 as its ligand. For instance, scholars have found that mtDNA releasing into the circulation by shock could activate neutrophil (PMN) p38 mitogen-activated protein kinase, probably *via* TLR9, inducing an innate-immune stimulatory “danger” response (12). Back in 2012, Oka et al. showed that mtDNA escaped from autophagy was capable of inducing myocarditis, and dilated cardiomyopathy *via* TLR9-mediated inflammatory responses in cardiomyocytes (10).

Taken together, mitophagy may be involved in the physiopathologic process of VILI, which highlights the interplay of inflammation and oxidative or endoplasmic reticulum (ER) stress between mitophagy and VILI. This manuscript intends to demonstrate whether HTV induce mitochondrial damage to activate mitophagy, resulting in mtDNA release. And to investigate the role and mechanism of mtDNA in VILI in rats.

## Materials and Methods

### Animals

Pathogen-free Sprague-Dawley rats were purchased from the Animal Center of Guangxi Medical University (Nanning, China), and approved by the Institutional Animal Care and Use Committee of Tumor Hospital of Guangxi Medical University. Rats were injected intravenously with 500 µg of CpG oligodeoxy nucleotides (ODN2088) 2.0 h before MV [tidal volume (VT) = 40 ml/kg] to inhibit the expression of TLR9 (10). Meanwhile, some rats were intraperitoneally pretreated with an inhibitor of mtDNA called chloroquine (CQ, 30 mg/kg) 2.0 h before MV [VT = 40 ml/kg] (13). EdU (5.0 mg/kg, 2.0 h/time and five times) was intraperitoneally injected to label mtDNA before MV (10). Antimycin A (AmA, 15 mg/kg, bw/day) and cyclosporine A (CsA, 5.0 mg/kg, bw/day) were intraperitoneally injected to inhibit mitochondrial electron transport and membrane permeability transition individually (14–16).

### Reagents

Enzyme linked immunosorbent assay (ELISA) kits were used to test the levels of inflammatory cytokines, including tumor necrosis factor-alpha (TNF-α), interleukin-6 (IL-6), interleukin-1 beta (IL-1β), and myeloperoxidase (MPO) (CUSABIO, Wuhan, China). DNase I (AMPD1), rat (I4131), and mouse IgG (I8765) were obtained from Sigma-Aldrich for the pretreatment of lung tissues; Percoll (P8370, Pharmacia), RPMI 1640-HEPES medium (22400097, Gibco), RT II-70 monoclonal antibody (gift from Professor Gonzalez, UCSF-Medical Center, San Francisco), goat anti-mouse IgG3 Secondary Antibody Alexa Fluor 488 conjugate (A-21151, Life Technologies) were used to isolate alveolar type II (AT-II) cells. Dulbecco’s modified Eagle’s medium (10567022, Gibco) with rat serum (D110-00-0050, Rockland), recombinant human keratinocyte growth factor (KGF/FGF-7, 251-KG-010, R&D Systems), 8-Bromoadenosine 3′, 5′-cyclic monophosphate (8-bromocyclic AMP, B5386, Sigma-Aldrich) and Engelbreth-Holmes-Swarm Matrix (EHS Matrix, Matrigel, 356234, BD) were employed for culturing AT-II cells. Then CQ and ODN2088 were purchased from Sigma-Aldrich (C6628) and InvivoGen (tlrl-2088), respectively. In addition, TRIzol (15596018, Invitrogen) and real time-quantitative polymerase chain reaction (RT-qPCR) kit (Takara) were used to test the TLR9, cytochrome *c* oxidase 4 (COX4), MyD88, nuclear factor (NF)-κB (NF-κB), and β-actin’s mRNA levels. LC3B (L7543, Sigma, 1:1,000), PINK1 (BC100-494, Novus biological, 1:1,000), Parkin (SAB4502077, Sigma-Aldrich, 1:800), Mfn1 (M6319, Sigma-Aldrich, 0.7 µg/ml), TLR9 (NBP1-76680, Novus, 1:1,000), MyD88 (4283, Cell Signaling Technology, 1:1,000), COX 4 (NB110-39115, Novus, 1:2,000), NF-κB (4764, Cell Signaling Technology, 1:1,200), and β-actin (4970, Cell Signaling Technology, 1:1,000) were used as primary antibodies and horseradish peroxidase (HRP)-conjugated mouse anti-rabbit antibody (7074, Cell Signaling Technology, 1:1,000) was used as secondary antibody in immunoblot and immunofluorescence assays. Moreover, ATP (MAK190, Sigma-Aldrich), ROS (MAK144, Sigma-Aldrich), and Δψm assay kits (V35116, Invitrogen) were used to evaluate the mitochondrial damage by MV. AmA and CsA were both obtained from Sigma (St. Louis, MO, USA) for regulation of mitophagy. An EdU from Click-iT EdU Alexa Fluor 488 Imaging Kit (C10337, Invitrogen) was applied to detect mtDNA in the section.

### MV Model and Sample Collection

The animal model was established successfully based on the previous study (7, 17). Briefly, rats were anesthetized by intraperitoneal injection of 60 mg/kg pentobarbital sodium and 80 mg/kg ketamine, and the rats were given with 15 mg/kg pentobarbital sodium every 30 min and 2 mg/kg/h pancuronium for muscle relaxation. After received 16-gauge tube by tracheotomy, all animals were allowed to breathe spontaneously or ventilated mechanically with room air (FiO_2_ = 0.21%) by a small animal ventilator (TOPO, Kent Scientific, Torrington, CT, USA). The ventilation rate was 80 breaths/min and the fraction of inspired oxygen was approximately at 40–50%. The inspiration to expiration ratio was kept at 1:1 throughout the experiment, no positive end expiratory pressure was included, and VT was calculated as previously described. After MV or spontaneous breathing, all rats were sacrificed by carotid artery bleeding, and the bronchoalveolar lavage fluid (BALF), blood serum, and lung tissue were collected and stored at −80°C except the right lung, which was obtained for paraffin embedding, transmission electron microscope (TEM) examining, and wet/dry (W/D) ratio calculating. It should be noted that all animal procedures were performed with great care to minimize activation of an inflammation.

### Inflammatory Responses

The W/D ratio was calculated to estimate the condition of lung edema during VILI. The middle lobe of right lung was weighed and then dried to a constant weight at 60°C for 48 h. Total protein of BALF was assessed for pulmonary permeability by bicinchonininc acid (BCA) assay, and cells were counted for inflammatory infiltration by hemocytometer. Moreover, IL-1β, IL-6, TNF-α, and MPO in plasma and BALF were detected by ELISA kits according to the manufacturer’s instructions.

### Histopathological Analysis

The right lower lung lobe was dissected and fixed with 10% formaldehyde. The lung tissues were embedded with paraffin and stained with hematoxylin and eosin. The degree of lung injury was estimated by scores according to four criteria: (1) alveolar congestion; (2) hemorrhages; (3) neutrophils’ infiltration; and (4) incrassation of the alveolar wall. Criterion were scaled as five-point scores: 0, minimal injury; 1, mild injury; 2, moderate injury; 3, serious injury; and 4, maximal injury. The cumulative histology score for all of the parameters was calculated, then the overall score of VILI was obtained. A mean ± SD was generated from the cohort of spontaneous breathing or ventilated lungs to generate a cumulative histological VILI (7, 18). Simultaneously, lung samples were cut for TEM analysis to observe lung cell epithelial cells and other cell injuries.

### RNA Extraction and RT-qPCR

Total RNA was isolated from lung tissue using TRIzol reagent. After RNA quality and quantity were determined by 260/280 nm absorbance, single-stranded cDNA was synthesized using the Takara RNA PCR kit. Total RNA was determined, and 1 µg of total RNA was reverse-transcribed into cDNA and amplified using SYBR Premix Ex Taq II and specific primers for TLR9, COX4, MyD88, NF-κΒ, and β-actin. The level of each target gene was normalized relative to that of β-actin in each sample using the ΔCt method. Relative differences in gene expression among groups of the lung tissues were determined using the comparative Ct (ΔΔCt) method and fold expression was calculated by the formula 2^−ΔΔCt^, where ΔΔCt represents ΔCt values normalized relative to the mean ΔCt of healthy control samples. The final data were shown as the ratio of the mean value of triplicate detection of the mtDNA samples (COX 4) and the average value of the nuclear gene (β-actin), namely mtDNA/β-actin (19).

### Immunoblot Analysis

Total protein was extracted, and the concentrations were assessed by BCA assay. Then, the molecular weight marker and each sample were added to the lanes of sodium dodecyl sulfate (SDS)-polyacrylamide gel. The proteins were transferred onto a nitrocellulose membrane and then blocked. Subsequently, the membranes were incubated by LC3B, PINK1, Parkin, Mfn1, COX 4, TLR9, MyD88, NF-κB, and β-actin primary antibodies and HRP-conjugated mouse anti-rabbit antibody as a secondary antibody. The bands of each protein from different samples were scanned and detected *via* a West Pico enhanced chemiluminescence kit (Thermo Fisher Scientific).

### Immunofluorescence Techniques

The slides from the paraffin-embedded lung tissues were dewaxed, hydrated, used for antigen retrieval, blocked, and cleared of endogenous peroxidases. Then, anti-LC3B was used as the primary antibody and anti-rabbit IgG (H + L), F(ab′)2 Fragment (Alexa Fluor 594 Conjugate) served as secondary antibody, along with DAPI for nuclear staining. Meanwhile, cells from the EdU-labeled rats were detected *via* Click-iT EdU Alexa Fluor 488 Imaging Kit according to the manufacturer’s directions to observe mtDNA accumulation in lung tissues, as well as for analyzing the source of escaped mtDNA. The slides were coated with Prolong Gold quenching resistant reagent and preserved at 4°C for imaging with fluorescence microscopy. The whole process was kept out of the sun.

### Cells Isolation and Purification

#### Alveolar Macrophages (AMs) Extraction

Alveolar macrophages were isolated and cultured according to the previous method (17). The collected BALF was centrifuged at 1,000 × *g* for 10 min and washed three times with pathogen-free PBS. The pellet was resuspended in RPMI 1640 media containing 10% fetal bovine serum (FBS) and 20 kU/l penicillin–30 kU/l streptomycin with 10% CO_2_ in air at 37°C for 3.0 h.

#### AT-II Cells Isolation

Dr. Gonzalez’s method (20) was used to isolate and culture AT-II cells. Briefly, the whitening lungs were minced by Mayo-Noble scissors and added to a 25 ml-beaker containing 20% FBS and 0.5 ml DNase I solution (2 mg/ml “Solution A”: RPMI 1640-HEPES medium). An additional 10 ml of “Solution A” was added to this beaker and the minced lung tissues with solution were transferred to a 250 ml-Erlenmeyer flask. Then, rat IgG was added to a final concentration of 50 µg/ml. The flask was shaken vigorously with 130 cycles/min for 2 min at reciprocating water bath, applying shear force to the lung minces. As the water bath shakes from side-to-side, the liquid should move back-and-forth rather than swirling. This liquid containing the lung minces was then filtered *via* 100, 40, and 20 µm nylon mesh and the cell suspension was transferred to a 50 ml-tube containing 50 µg/ml mouse IgG. Moreover, 1 ml of RT II-70 was added to this cell suspension and incubated for 10 min on ice, followed by centrifugation at 350 × *g* for 12 min at 4°C with 150 µl of Percoll cushion. The resulting pellet was resuspended with 50 µl of DNase I and 1 ml of “Solution A” in 20% FBS to a 1.3 ml total volume. 0.3 ml of the cell suspension was aliquot into three tubes, and to each 0.1 µl of RT II-70, 0.1 µl of PBS and 0.5 µl of goat anti-mouse IgG_3_ secondary antibody Alexa Fluor^®^ 488 conjugate with 0.5 µl of RTII-70 was added. In addition, the remaining 1 ml of cell suspension was added 5 µl of goat anti-mouse IgG3 secondary antibody Alexa Fluor 488 conjugate and 5 µl of RTII-70 for cell isolation. Both of these tubes were incubated for 10 min at room temperature and diluted into 15 ml/tube with “Solution A.” These tubes were then added to 150 µl Percoll cushion on the bottom of each tube and centrifuged at 350 × *g* for 15 min at 4°C. The pellet was resuspended with 50 µl of DNase I and 0.5 ml of “Solution A” in 20% FBS for further flow cytometer sorting. AT-II cells were cultured in Dulbecco’s modified Eagle’s medium with 1% rat serum, 10 ng/ml KGF, 10^−4^ M 8-bromocyclic AMP, and 20 kU/l penicillin–30 kU/l streptomycin on an EHS matrix with 10% CO_2_ in air at 37°C.

### Mitochondrial Damage Evaluation

The MV-induced mitochondrial damage with mitophagy was evaluated *via* measuring ATP levels, ROS production, and Δψm. The ATP level, ROS production, and Δψm were assayed using corresponding assay kits from Sigma-Aldrich according to the manufacturer’s instruction. Briefly, the ATP levels were determined *via* firefly luciferase-associated chemiluminescence and ROS production was detected by 2′7′-dichlorofluorescin diacetate using flow cytometry analysis. The Δψm was observed using a fluorescent probe JC-1 that assembles as J-aggregates with red fluorescence in the mitochondrial matrix during higher Δψm, but is depolymerized as monomer and results in green fluorescence.

### DNase-II Expression

The single-phase enzyme diffusion (21) method was used to compare DNase-II expression in the serum. In brief, “Solution A” [1 ml of 6 g/l calf thymus DNA; 50 µl of 10 g/l ethidium bromide; pH 7.2, 0.05 M Tris–HCl (0.05 M MgCl_2_) 8~10 μl; 40 µl of 1 M CaCl_2_] and “Solution B” (10 ml of 2% agarose in diethyl pyrocarbonate water) were mixed to make agar board which was then bored with a 1.5 mm-diameter puncher. 4 µl of either serum samples or standard DNase-II were added to the agar board holes, followed by 36 h of incubation at 37°C. The agar board was then soaked with 0.1 M elhylene diamine tetraacetic acid (EDTA) to stop the reaction and the diameter of DNA hydrolyzed loop was measured using ultraviolet radiation.

### mtDNA Extraction and Purification

The extraction and purification of mtDNA were modified from the previous study to improve the yield and quality of mtDNA (22). Briefly, a pellet composed of 10^7^ cells were resuspended with 5.5 ml “Solution I” of (10 M Tris–HCl; 10 M NaCl; 5 M MgCl_2_; pH = 7.5) and centrifuged at 2,000 rpm for 10 min. The pellet was resuspended in 5.5 ml of “Solution II” (10 mM Tris–HCl; 0.4 M NaCl; 2 mM EDTA; pH = 7.5) and centrifuged at 3,500 rpm for 10 min. Then the pellet was resuspended in 0.95 ml of “Solution II” and 50 µl of 20 mg/ml protease K and 10% SDS were added, followed by incubation at 4°C overnight. 0.3 ml of saturated sodium acetate was added to the suspension liquid and lightly shaken, followed by centrifugation at 15,000 rpm for 15 min at 4°C. The supernatant was obtained, and the last step was repeated. A phenol:chloroform:isopropanol (25:24:1) solution was added to the supernatant, agitated by light shaking and centrifuged at 10,000 rpm for 10 min at 4°C. The upper water phase was transferred to a new tube, and the previous step was repeated. Two volumes of ice-cold ethanol were added to the upper water phase and incubated on ice for 30 min, followed by centrifugation at 15,000 rpm for 10 min at 4°C. 70% ice-cold ethanol was next used to wash the pellet, centrifuging the sample at 15,000 rpm for 2 min at 4°C. The pellet was dried for 25 min at room temperature and finally dissolved by recombinational trypsin/EDTA solution at storage of 4°C.

### Statistical Analysis

The analyses of data were conducted by SPSS 13.0 software. All quantitative data were demonstrated as mean ± SD. One-way ANOVA was operated in order to analyze multiple comparisons of each groups, followed by the LSD-*t* test and the SNK test for pair-wise comparisons. Chi-square test was used for analysis of categorical variables. The statistical difference was defined by *P* value less than 0.05.

## Results

### MV With HTV Induces Lung Injury and Inflammation

**Figure 1:**
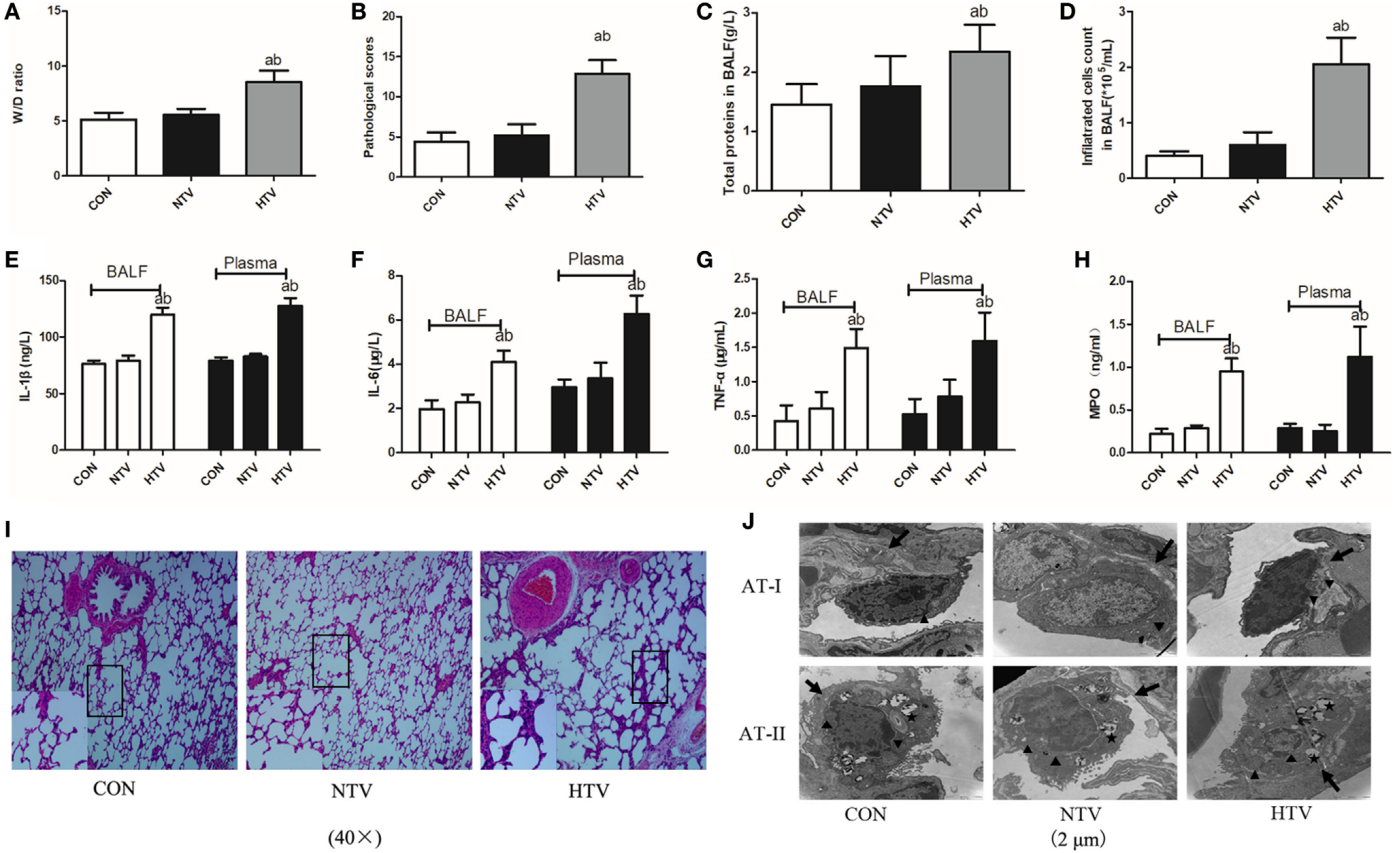
Lung injury and inflammation in animals with spontaneous breathing (CON) or mechanical ventilation at normal tidal volume (NTV) or high tidal volume (HTV). **(A)** Lung edema was assessed by determining the weight ratio between wet and dry lung (W/D). **(B)** Pathological scores were assessed by results of hematoxylin and eosin (HE) staining. **(C)** Total protein concentration in bronchoalveolar lavage fluid (BALF). **(D)** Infiltrated cell counts in BALF. **(E)** Levels of interleukin (IL)-1β in BALF and plasma. **(F)** Levels of IL-6 in BALF and plasma. **(G)** Levels of tumor necrosis factor (TNF)-α in BALF and plasma. **(H)** Levels of myeloperoxidase (MPO) in BALF and plasma. **(I)** Histology of lung tissue was stained with HE. Magnification, 40×. **(J)** Transmission electron micrographs of alveolar cells. Areas marked with arrows signify continuous membrane and triangles mark areas of disrupted cytoplasmic and nuclear structure. Areas marked with stars represent the osmilphilic multilamellar bodies, the unique structure of AT-IIs. Tissue from CON and NTV groups appeared normal, but tissue from HTV group exhibited a disrupted cytoplasmic and nuclear structure, as well as cell membrane discontinuities. In addition, the osmilphilic multilamellar bodies show characteristic vacuolation in the HTV group. Magnification, 20,000×. Both of these experiments were in triplicate. ^a^*P* < 0.05 vs. CON group; ^b^*P* < 0.05 vs. NTV group.

Anesthetized animals were randomly allocated into three groups (*n* = 12) using table of random number: non-ventilated (CON), ventilated with normal VT (10 ml/kg, NTV), and ventilated with high VT (40 ml/kg, HTV). All animals survived the 4-h period of spontaneous breathing or MV at normal or high VT. The rats treated with high VT exhibited significantly severe pulmonary edema and higher BALF total protein levels than the rats treated with spontaneous breathing and normal VT by determining the lung W/D ratios (Figures 1A,C). The lung histopathology score was higher in high VT rats as compared with the CON and NTV group. However, no differences were noted between the CON and NTV group (Figure 1B). In the HTV group, significantly more cells were infiltrated than in the NTV group and non-ventilated animals (Figure 1D). In addition, the levels of cytokine profiles, including IL-1β, IL-6, TNF-α, and MPO, in BALF and plasma were significantly higher in the HTV group than that in the CON and NTV groups. And these cytokine profiles were similar in animals ventilated with normal VT and control animals (Figures 1E–H).

Lungs from animals ventilated with high VT showed acute inflammatory infiltration, perivascular edema, and more alveolar septal thickening, whereas no major histological differences were observed between animals ventilated with normal VT and spontaneous breathing control animals (Figure 1I). Using TEM to examine alveolar histopathology in greater detail, we found that, as expected, alveolar cells in the tissue of the rats ventilated with high VT exhibited a disrupted cytoplasmic and nuclear structure, as well as cell membrane discontinuities. However, tissue from rats ventilated with normal VT and spontaneous breathing control animals showed a normal cytoplasmic and nuclear structure and continuous cell membrane for types I and II alveolar epithelial cells. The osmilphilic multilamellar body is the unique structure of AT-IIs. In addition, the osmilphilic multilamellar bodies show characteristic vacuolation in the AT-IIs of HTV group (Figure 1J).

### MV With HTV Induced TLR9 Expression and Upregulated Protein Levels of MyD88 and NF-κB

**Figure 2:**
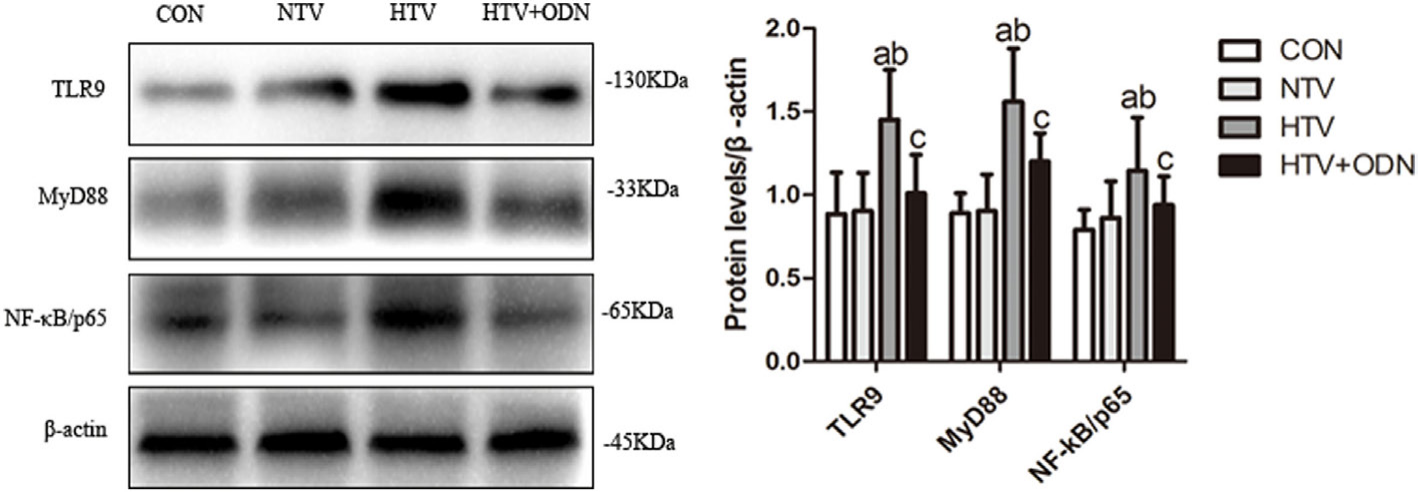
Levels of toll-like receptor (TLR) 9, myeloid differentiation factor 88 (MyD88), and nuclear factor (NF)-κB in lung tissues from animals with spontaneous breathing (CON), mechanical ventilation at normal tidal volume (NTV), high tidal volume (HTV), and ODN2088 pretreatment upon HTV stimulation (HTV + ODN). ^a^*P* < 0.05 vs. CON group;^b^ *P* < 0.05 vs. NTV group; and ^c^*P* < 0.05 vs. HTV group.

The protein levels of TLR9, MyD88, and NF-κB were significantly higher in the HTV group as compared with the other groups. Moreover, pretreated with ODN2088 could significantly ameliorated the HTV-induced these proteins in the lungs. It might suggest that HTV induces inflammatory response in the lungs *via* TLR9/MyD88/NF-κB signaling pathway (Figure 2).

### MV With HTV Favors the Damage of Mitochondrial and the Release of mtDNA

**Figure 3:**
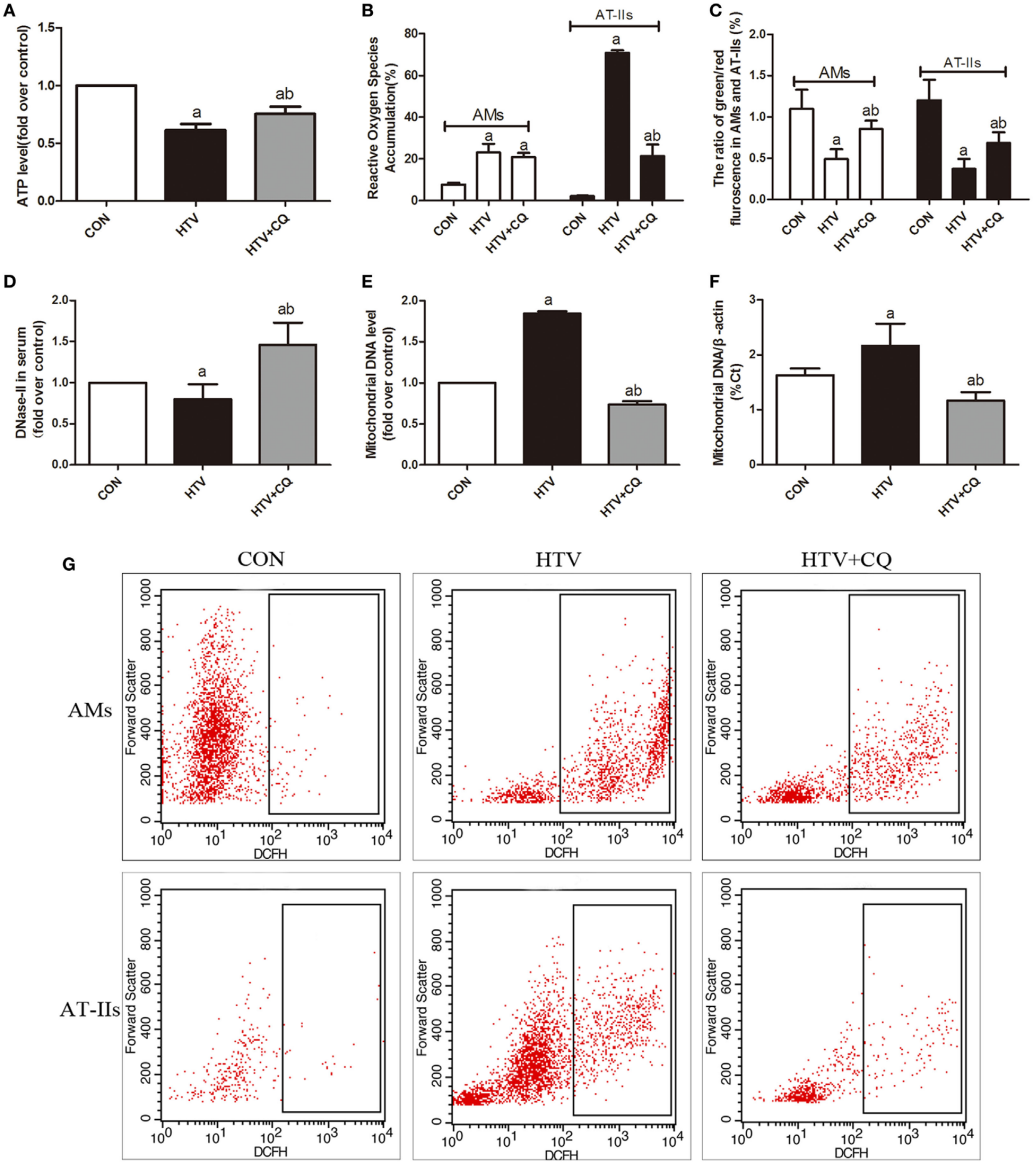
Mechanical ventilation with high tidal volume (HTV) favors the damage of mitochondrial and the release of mitochondrial DNA (mtDNA). **(A)** Levels of ATP. **(B)** Levels of reactive oxygen species (ROS) in alveolar macrophages (AMs) and AT-IIs. **(C)** Levels of Δψm were noted by the decreased ratio of green (JC-1 monomer for low Δψm)/red (J-aggregates for high Δψm) fluorescence in AMs and AT-IIs. **(D)** Levels of DNase-II in plasma. **(E)** Levels of isolated mtDNA (fold over control). **(F)** Relative levels of mtDNA by measurement of the β-actin gene. **(G)** The flow cytometry analysis of ROS. Both of these experiments were in triplicate. ^a^*P* < 0.05 vs. CON;^b^ *P* < 0.05 vs. HTV group.

In order to explore whether MV with HTV can favor the damage of mitochondrial and the release of mtDNA, ATP levels, ROS production, Δψm variation, and expression level of mtDNA were measured. The level of ATP in the HTV was decreased in comparison to the rats treated with spontaneous breathing and pretreated CQ could alleviate the result (Figure 3A). Then, the production of ROS in HTV group was significantly increased in comparison to the rats treated with spontaneous breathing, but pretreated with CQ could reduce these ROS induced by MV with HTV, especially in AT-IIs (Figures 3B,G). Moreover, the Δψm in the HTV group was decreased in comparison to the rats treated with spontaneous breathing, while compared with the animals over-ventilated with CQ (Figure 3C).

The level of serum DNase-II (a main enzyme for degradation of mtDNA) in the HTV was decreased in comparison to the rats treated with spontaneous breathing or normal VT (Figure 3D). The outcome of RT-qPCR in isolated mtDNA in the HTV group was significantly higher than that in CON group, but level of mtDNA was significantly lower in the CQ pretreatment group compare with the HTV group (Figures 3E,F).

### Inhibition of COX4 Regulates the Expression of COX4, TLR9, MyD88, and NF-κB

**Figure 4:**
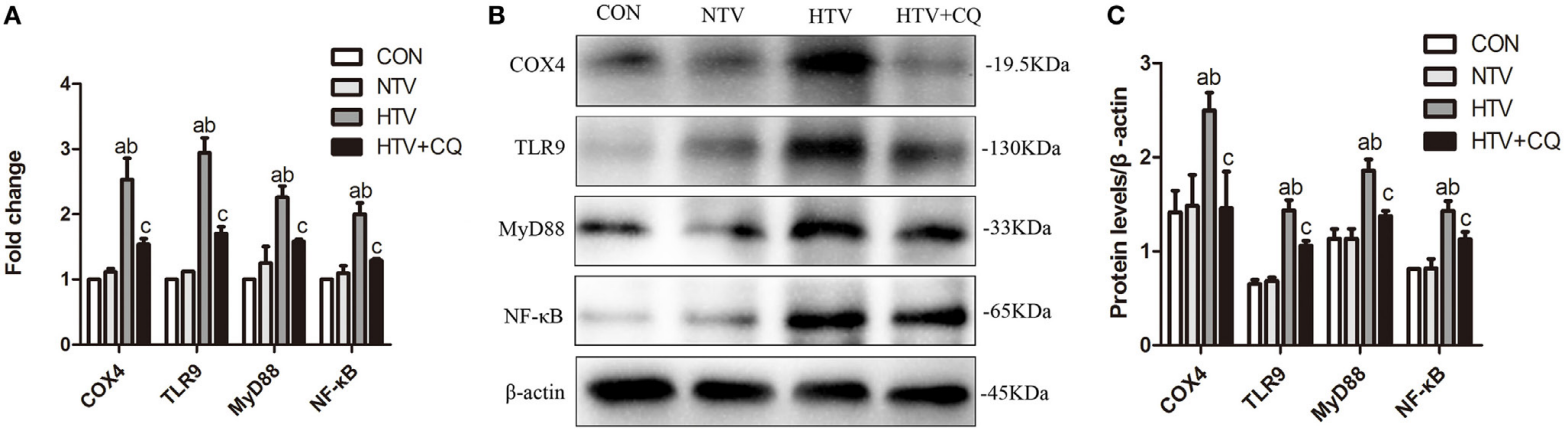
The mRNA and protein expression of cytochrome *c* oxidase 4 (COX4), toll-like receptor (TLR) 9, myeloid differentiation factor 88 (MyD88), and nuclear factor (NF)-κB in lung tissues from spontaneous breathing group (CON group), normal tidal volume (NTV) group, high tidal volume (HTV) group, chloroquine (CQ) pretreatment upon HTV stimulation group (HTV + CQ). **(A)** Levels of COX4, TLR9, MyD88, and NF-κB mRNA. **(B)** Levels of COX4, TLR9, MyD88, and NF-κB protein by Western blot. **(C)** Relative expression of COX4, TLR9, MyD88, and NF-κB protein. Fold expression for target genes was normalized to that measured for the β-actin gene. Both of these experiments were in triplicate. ^a^*P* < 0.05 vs. CON group; ^b^*P* < 0.05 vs. NTV group; and ^C^*P* < 0.05 vs. HTV group.

The mRNA and protein levels of COX4, TLR9, MyD88, and NF-κB were significantly higher for animals ventilated with high VT than animals in the CON and NTV groups. Expectedly, after pretreated with mtDNA inhibitor CQ, the mRNA and protein expression of COX4, TLR9, MyD88, and NF-κB in animals treated with high VT were significantly inhibited (Figures 4A–C).

### mtDNA Escaped From a Selective Autophagy Named Mitophagy During MV With HTV

**Figure 5:**
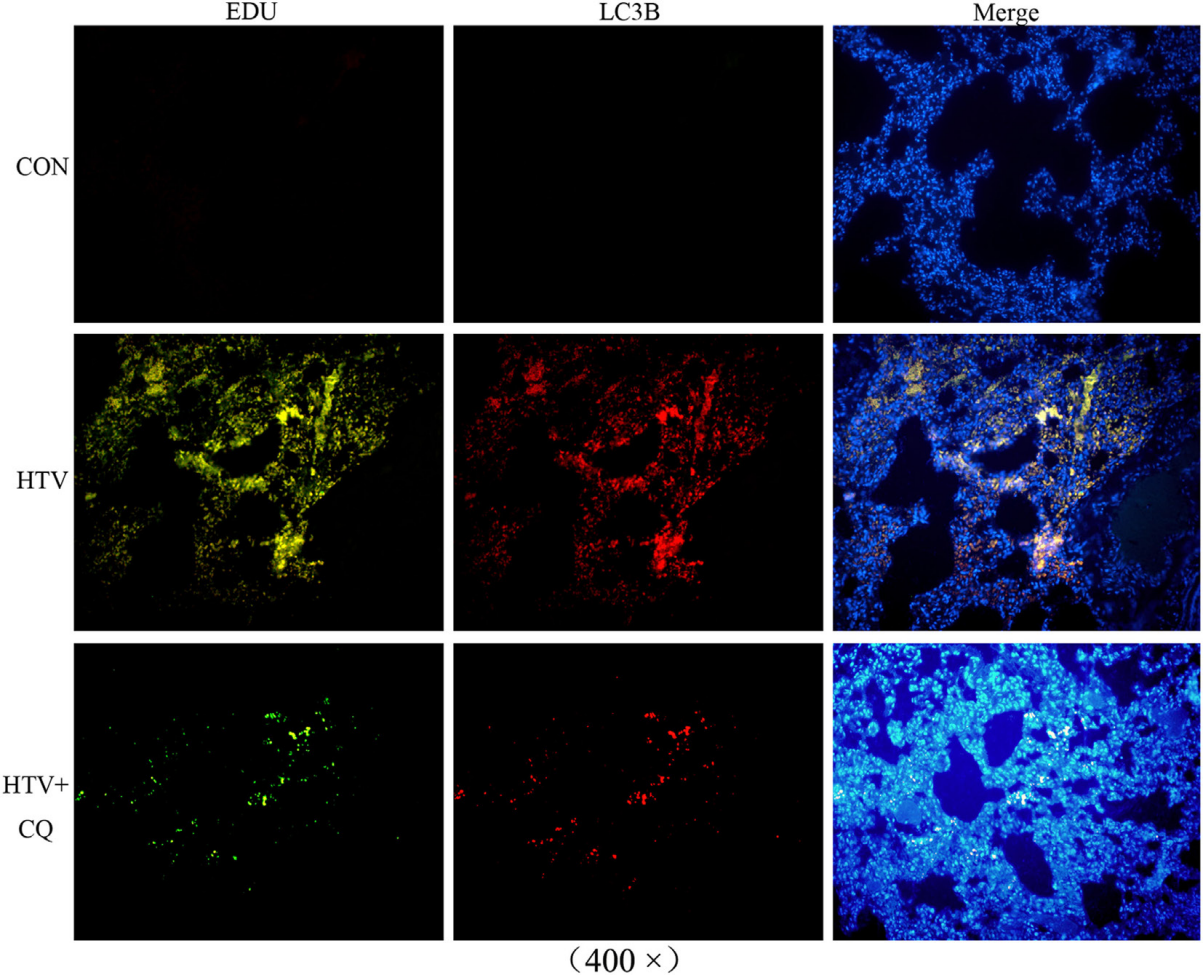
Immunofluorescence studies of EdU-labeled mitochondrial DNA (mtDNA) (green) and crucial autophagic protein LC3B (red) in lung tissues from animals with spontaneous breathing (CON) and mechanically ventilated rats pretreated with saline [high tidal volume (HTV)], CQ (HTV + CQ). Immunofluorescence studies confirmed that the EdU-labeled mtDNA (green) was similarly distributed and accumulated with the crucial autophagic protein LC3B, which was labeled with Alexa Fluor 594 (red). Magnification, 400×. Both of these experiments were in triplicate.

The expression of LC3B conjugated with a fluorescence moiety was recommended in order to observe the autophagy state and the EdU was used for mtDNA distribution. The fluorescence of mtDNA and LC3B, which specifically marked mitophagy, turned up in same places, indicating that mtDNA could be released due to autophagic lysosomes. The fluorescence intensity showed that the expression of LC3B and mtDNA in the HTV was increased compared to CON group. More importantly, the expression of LC3B and mtDNA in the HTV + CQ group was lower than that in the HTV group (Figure 5).

### Regulation of Mitophagy Plays an Important Role in VILI *via* TLR9–MyD88–NF-κB Pathway

**Figure 6:**
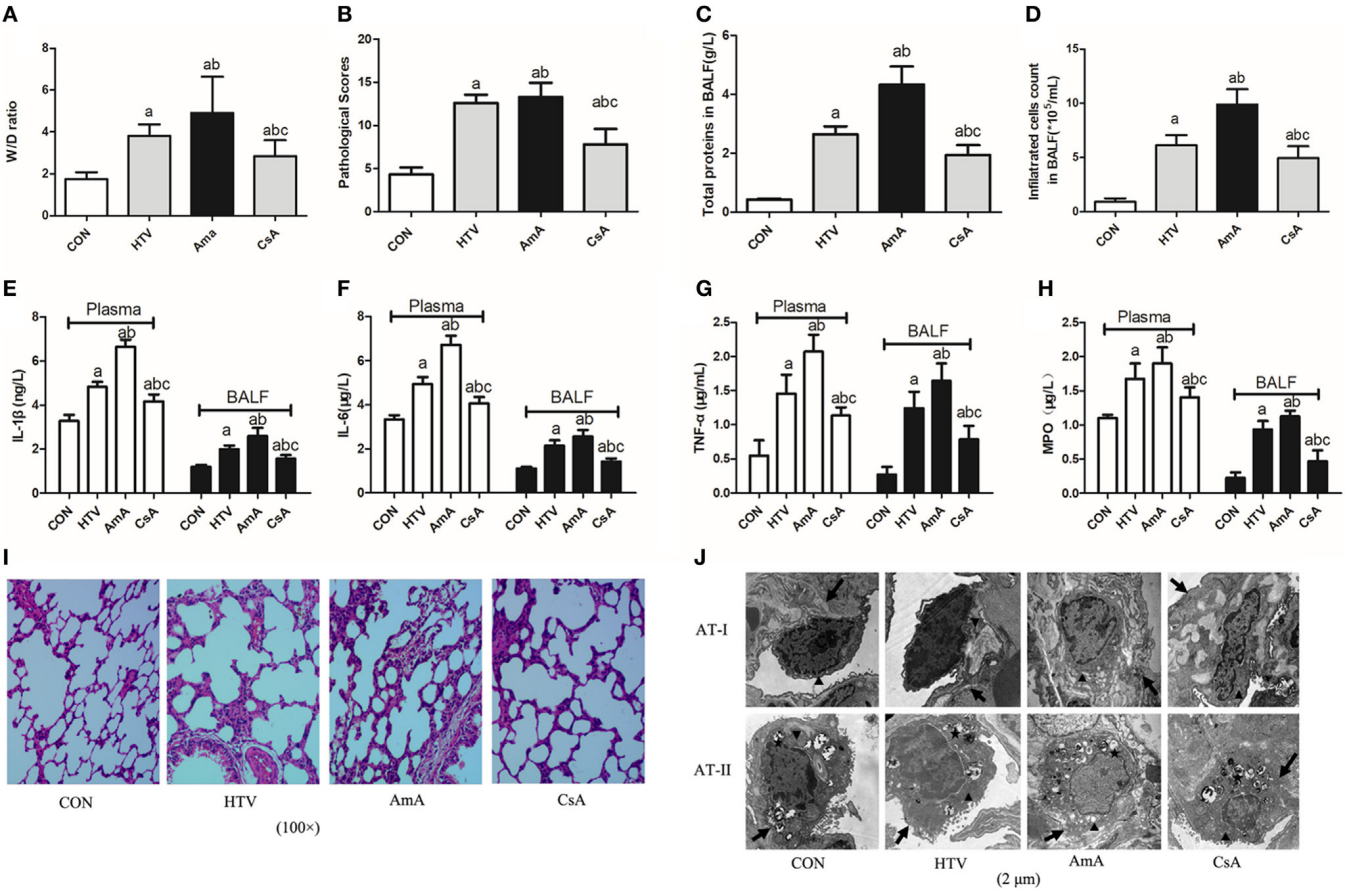
Lung injury and inflammation in animals with spontaneous breathing (CON) or mechanical ventilation at high tidal volume (HTV) with saline, antimycin A (AmA), or cyclosporine A (CsA). **(A)** Lung edema was assessed by determining the weight ratio between wet and dry lung (W/D). **(B)** Pathological score was assessed by result of hematoxylin and eosin (HE) staining. **(C)** Total protein concentration in bronchoalveolar lavage fluid (BALF). **(D)** Infiltrated cell counts in BALF. **(E)** Levels of interleukin (IL)-1β in BALF and plasma. **(F)** Levels of IL-6 in BALF and plasma. **(G)** Levels of tumor necrosis factor (TNF)-α in BALF and plasma. **(H)** Levels of myeloperoxidase (MPO) in BALF and plasma. **(I)** Histology of lung tissue was stained with HE. Magnification, 100×. **(J)** Transmission electron micrographs of alveolar cells. Areas marked with arrows signify continuous membrane and triangles mark areas of disrupted cytoplasmic and nuclear structure. Areas marked with stars represent the osmilphilic multilamellar bodies, the unique structure of AT-IIs. Tissue from CON group appeared normal, but tissue from HTV and AmA groups exhibited a disrupted cytoplasmic and nuclear structure, as well as cell membrane discontinuities. Moreover, the osmilphilic multilamellar bodies show characteristic vacuolation in the HTV and AmA groups. But these effects were attenuated when administered in the presence of CsA. Magnification, 20,000×. Both of these experiments were in triplicate. ^a^*P* < 0.05 vs. CON group;^b^ *P* < 0.05 vs. HTV group; and ^c^*P* < 0.05 vs. AmA group.

The role of mitophagy in VILI was shown by presence of antimycin A (inhibitor of mitochondrial electron transport) and cyclosporine A (inhibitor of mitochondrial membrane permeability transition). AmA pretreatment aggravated lung edema (Figure 6A) and morphological injuries (Figures 6B,I,J), but CsA pretreatment attenuated the lung edema and injury. Total protein level for pulmonary permeability in BALF was significantly increased in the AmA group compared to the HTV group and CON group (Figure 6C), while infiltrated cells counted in BALF was consistent with the variation of total proteins (Figure 6D). The concentrations of inflammatory factors, including IL-1β, IL-6, TNF-α, and MPO, in the AmA group (plasma and BALF) were higher than those in the HTV and control groups, and CsA pretreatment could attenuate these inflammatory factors (Figures 6E–H).

**Figure 7:**
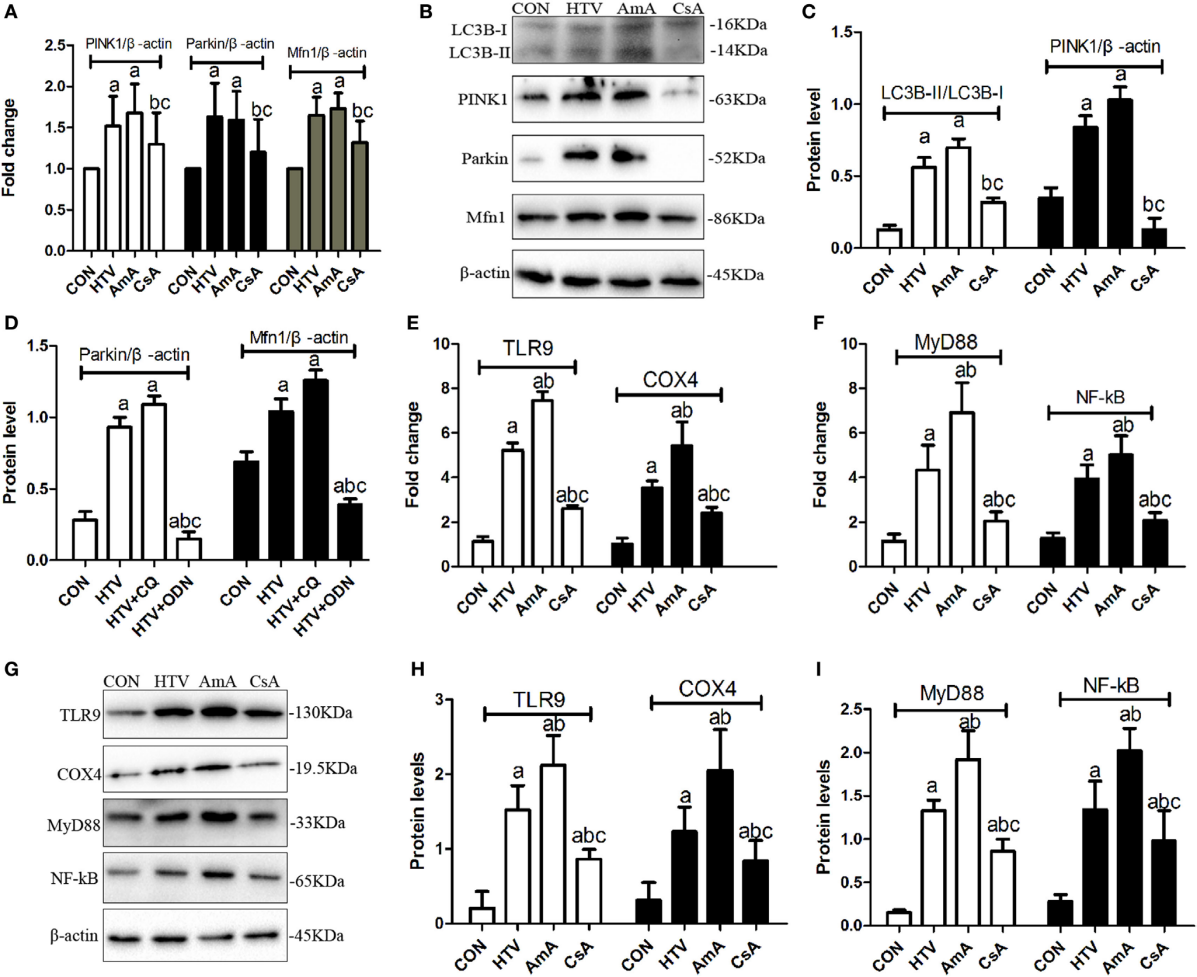
The expression levels of microtubule protein light chain 3 (LC3B), PTEN inducing putative kinase 1 (PINK1), Parkin, mitofusin 1 (Mfn1), toll-like receptor (TLR) 9, cytochrome *c* oxidase 4 (COX4), myeloid differentiation factor 88 (MyD88), and nuclear factor (NF)-κB in lung tissues from animals with spontaneous breathing (CON) or mechanical ventilation at high tidal volume (HTV) with saline, antimycin A (AmA) or cyclosporine A (CsA). **(A)** Levels of PINK1, Parkin, and Mfn1 mRNA. **(B)** Levels of LC3B, PINK1, Parkin, and Mfn1 protein by Western blot. **(C)** Relative expression of LC3B-II/LC3B-I and PINK1 protein. **(D)** Relative expression of Parkin and Mfn1 protein. **(E)** Levels of TLR9 and COX4 mRNA. **(F)** Levels of MyD88 and nuclear factor-κB (NF-κB) mRNA. **(G)** Levels of TLR9, COX4, MyD88, and NF-κB protein by Western blot. **(H)** Relative expression of TLR9 and COX4 protein. **(I)** Relative expression of MyD88 and NF-κB protein. Fold expression for target genes was normalized to that measured for the β-actin gene. Both of these experiments were in triplicate. ^a^*P* < 0.05 vs. CON group;^b^ *P* < 0.05 vs. HTV group; and ^c^*P* < 0.05 vs. AmA group.

The expression levels of mitophagy related genes (LC3B-II/LC3B-I, PINK1, Parkin, and Mfn1) in animals ventilated with high VT were significantly upregulated (Figures 7A–D). AmA favored the higher ratio of LC3B-II/LC3B-I, expression of PINK1, Parkin, and Mfn1, while CsA inhibited the expression of these mitophagy related proteins (Figures 7A–D). What is more, AmA pretreatment upregulated the mRNA and protein level for COX4, TLR9, MyD88, and NF-κB, but CsA pretreatment attenuated the expression of these genes (Figures 7E–I).

## Discussion

Mechanical ventilation is life saving during the perioperative period, but inappropriate MV can lead to the development of VILI (23, 24). VILI is the result of a complex systematic inflammation associated with release of various proinflammatory mediators and activation of inflammatory signaling pathways (25). A better understanding of the mechanisms of VILI in lungs is needed if protective ventilator strategies are to be developed further.

In this study, rats subjected to HTV developed marked pathologic changes, ultrastructure changes in alveolar and release of various inflammatory cytokines (Figure 1). Pretreatment with ODN2088 significantly ameliorated the phenomenon HTV-induced described above, which was further explored that HTV ventilation caused the release of various mediators *via* TLR9/MyD88/NF-κB signaling pathway, leading to VILI in rats (Figure 2). The result is in accordance with our previous results (7). Meanwhile, MV with HTV damaged mitochondria, causing dysfunctional ATP synthesis (Figure 3A), accumulation of ROS (Figures 3B,G) and loss of Δψm (Figure 3C), especially in AT-IIs. The level of DNase-II and isolated mtDNA revealed that MV with HTV could cause the release of mtDNA (Figures 3D–F). Moreover, pretreatment with CQ reduced the protein and mRNA levels of COX4, TLR9, MyD88, and NF-κB (Figure 4). These findings suggested that mtDNA (COX4) may, at least in part through TLR9/MyD88/NF-κB signaling pathway, initiate an immunological response characteristic of VILI.

Interestingly, HTV-induced accumulation of ROS was observed in AT-IIs rather than AT-Is in the present study. AT-Is cover approximately 90–95% of the alveolar surface and involve in the process of gas exchange between the alveoli and blood; AT-IIs favor the secretion of pulmonary surfactant and differentiation to AT-Is. At the cellular level, AT-IIs play the more important role in regulation of inflammation, apoptosis, oxidative, and ER stress during MV with HTV (26, 27). It showed that pulmonary autophagy or mitophagy mainly occurred in macrophages and AT-IIs during MV, *Mycobacterium tuberculosis*, and *S. aureus* infection (10, 28, 29).

In order to confirm the possible release of mtDNA during MV with HTV, immunoblot analysis (Figure 7B) and immunofluorescence (Figure 5) were measured. Results indicated that MV with HTV could activate mitophagy, causing damaged mitochondria to engulf as a result of mtDNA release from damaged mitochondria. Previous researches have shown that increased ROS and reduced ATP levels can be accompanied with mtDNA damage, NF-κB and MyD88 upregulation, triggering inflammation through the TLR9 pathway in cells and mouse models (9, 30), which was consistent with our studies described above.

In the current study, the role of mitochondrial damage in VILI was shown by presence of antimycin A (inhibitor of mitochondrial electron transport) and cyclosporine A (inhibitor of mitochondrial membrane permeability transition) (Figures 6 and 7). AmA pretreatment aggravated lung injuries, favored the higher ratio of LC3B-II/LC3B-I, expression of PINK1, Parkin, and Mfn1, and upregulated the mRNA and protein level of COX4, TLR9, MyD88, and NF-κB. But CsA pretreatment showed opposite results. The results indicated that regulation of mitophagy plays an important role in VILI.

Autophagy is a cytoprotective process that is activated for the turnover and metabolism of proteins, as well as in eliminating damaged organelles or pathogens (31, 32). Mitophagy, a selective autophagy, was activated to remove damaged mitochondria and maintain mitochondrial quality control. The recruitment of damaged mitochondria to the autophagosome is initiated by the phosphatase and tensin homolog deleted in chromosome 10-induced PINK1 that is stabilized on depolarized or damaged mitochondria *via* Parkin (33). After loss of Δψm, PINK1 recruits Parkin from the cytosol to the mitochondria, at which point it interacts with the GTPase Mfn, and then Parkin polyubiquitates the chains that marks the depolarized mitochondria for degradation. Parkin ubiquitinates many mitochondrial outer membrane proteins, such as porin, Mfn, and Miro, which are subsequently recognized and combined with the autophagic cargo adaptor protein p62 to deliver to autophagosomes (34). Confronted with oxidative stress, starvation, and pathogens, mitophagy is activated to eliminate redundant or damaged components, consequently causing inflammation. Mitophagy in Atg5 deficiency model facilitate cardiac inflammation and injury in response to angiotensin II (35).

Mitochondria DAMPs induce “sepsis-like” inflammation and mediate organ damage. It reported that mitochondrial DAMPs triggered inflammation and recruited inflammatory factors, including TNF-α, IL-6, and IL-10, under hepatic ischemia/reperfusion injury (36). Mitochondrial DAMPs act as potential proinflammatory mediators that participate in innate cell inflammation of non-hemolytic transfusion reactions (37). Moreover, mitochondrial DAMPs in circulation are major arbiters for the systematic inflammatory response syndrome during trauma (38). But there was study demonstrated that mitochondrial DAMPs released from damaged tissues by trauma also suppress immune responses (39). Finally, combining with our study, results indicate that released mtDNA could act as DAMPs to mediate inflammation during MV.

The released mtDNA was recently considered to be mitochondrial DAMPs to induce inflammation. Mitochondrial damage and mtDNA release is not favored the inflammation in myocardium, atherosclerosis (40), and pulmonary diseases (41), but is triggered during the ER stress and autophagy induced by palmitate in skeletal muscle cells (42). Moreover, mitochondria are involved in initiating inflammasomes and inflammatory pathways, including excess ROS generation that can result in mtDNA mutations to initiate a vicious cycle of mitochondrial collapse (43), as well as activating the NLRP3 inflammasome, the adapter protein ASC, and caspase-1 (44). What is more, some studies have shown that mtDNA, containing unmethylated CPG motifs, which can be recognized by intracellular TLR9, has been shown to have powerful immunostimulatory effects (10, 12, 45, 46). Furthermore, TLR9 regulated by mtDNA can activate NF-κΒ signaling, which thereby induces transcription of many proinflammatory cytokines levels, such as TNF-α, IL-6, and IL-1β (12, 47). These data are consistent with the results of our present study.

### Limitations

Although mtDNA is clearly involved in inflammation through the TLR9/MyD88/NF-κB pathway in regulating inflammatory cytokines during MV, there are several limitations that warrant further discussion. First of all, there are some other factors can affect injury severity, such as positive end expiratory pressure and variations in pressure support (48). For minimizing these impacts, we continuously monitored hemodynamic stability and oxygen saturation in the anesthetized rats. Second, according to the literature, the type and dose of anesthetic (e.g., sevoflurane, ketamine, or protofol) use can also affect animal studies on acute lung injury (49, 50). In addition, our study, however, were done only in living animals. We cannot say whether the results would be the same *in vitro* experiments.

## Conclusion

Excessive MV with HTV triggers mitochondria damage to activate mitophagy, resulting in mitochondrial membrane fracturing and mtDNA release, which was recognized by TLR9 that modulate inflammatory factor expression through TLR9/MyD88/NF-κB signaling pathway during VILI.

## Ethics Statement

This study was carried out in accordance with the recommendations of “the Animal Center of Guangxi Medical University (Nanning, China).” The protocol was approved by the “Institutional Animal Care and Use Committee of Tumor Hospital of Guangxi Medical University.”

## Author Contributions

L-hP designed and directed the overall study. J-YL and RJ carried out the main experiments, contributed to the collection and analysis of data, and wrote the paper. FL, H-jD, and W-yG carried out experiments and collected and analyzed data with RJ. All authors read and approved the final manuscript.

## Conflict of Interest Statement

The authors declare that the research was conducted in the absence of any commercial or financial relationships that could be construed as a potential conflict of interest.
